# *Coxiella burnetii* Nine Mile phase I primary infection derived protective immunity against C. *burnetii* reinfection in mice depends on both B and T cells, but T cells play a critical role

**DOI:** 10.3389/fimmu.2024.1427822

**Published:** 2024-10-14

**Authors:** Shawkat Alam, Venkatesh Kumaresan, Rajesh Palanisamy, Yan Zhang, Janakiram Seshu, Na Xiong, Guoquan Zhang

**Affiliations:** ^1^ Department of Molecular Microbiology and Immunology, The University of Texas at San Antonio, San Antonio, TX, United States; ^2^ Department of Microbiology, Immunology and Molecular Genetics, The University of Texas Health Science Center San Antonio, San Antonio, TX, United States

**Keywords:** *Coxiella burnetii*, Q fever, infection, protective immunity, B cells, T cells, antibodies

## Abstract

*Coxiella burnetii* is an obligate intracellular Gram-negative bacterium that causes acute and chronic Q fever in humans. Acute Q fever is usually a flu-like, self-limiting or treatable illness, but some infections can turn into a severe and sometimes fatal chronic disease. There is currently no FDA-approved vaccine available for the prevention of human Q fever in the US, development of a safe and effective vaccine for the prevention of human Q fever remains an important goal for public health. However, there is a fundamental gap in knowledge regarding the mechanism of protective immunity against *C. burnetii* infection. To understand the mechanism of *C. burnetii* infection induced protective immunity, we examined if *C. burnetii* Nine Mile phase I (NMI) infection induces protection against *C. burnetii* reinfection in mice. Our results indicate that NMI-infected mice conferred significant protection against *C. burnetii* reinfection. We also found that NMI infection derived protection did not depend on the routes of infection and antibodies are required for NMI infection derived protection. In addition, NMI infection elicited a comparable level of protection in Wild type, CD4^+^ T cell deficient, and CD8^+^ T cell deficient mice, partial protection in B cell deficient mice but no protection in T cell deficient mice. These results suggest that both B cells and T cells are required for NMI-infection derived protection, but T cells may play a critical role. Therefore, the new generation vaccine for the prevention of human Q fever should be focused on boosting both humoral and T cell immune responses.

## Introduction

*Coxiella burnetii* is an obligate intracellular Gram-negative bacterium that causes the worldwide zoonosis Q fever in humans. It typically spreads via aerosols generated by infected domestic animals, such as cattle, sheep, and goats (1). Acute Q fever exhibits a flu-like illness with fever, chills, fatigue, headache, and body aches. The acute infection can be asymptomatic and the most of cases are self-limiting (2). Consequently, there is a common belief that disease incidence is significantly underreported (3). Chronic Q fever commonly exhibits as an endocarditis and more than 25% of patients are fatal if left without specific treatments (1). Treatment of chronic Q fever patients typically involves dual antibiotic therapy with combination of doxycycline and hydroxychloroquine for at least 18 months (4). However, in a cohort study for 24 months indicated that more than 30% of Q fever patients retained an impaired health status despite following the antibiotic treatment (5). Since *C. burnetii* natural infections in humans commonly occur via inhalation of aerosols generated from *C. burnetii* infected ruminants, it is considered as an occupational hazard for individuals who are closely working with livestock (6). *C. burnetii’s* hardiness in the environment, transmission via respiratory route, and the low infectious dose made this organism a dangerous zoonotic pathogen. Furthermore, *C. burnetii* has been designated as a Tier 2 select agent by the Centers for Disease Control (CDC) due to its potential to be used as a biological warfare agent (7). Considering the incapacitating effects of aerosolized *C. burnetii* and the shortcomings of current antibiotic therapies, creating a safe and effective Q fever vaccine is an urgent and important goal for both public health and biosecurity. A formalin-inactivated whole-cell vaccine that generated from *C. burnetii* Henzerling phase I (WCV, also known as Q-VAX) has demonstrated to be able to elicit long-lasting protective immunity in animal models and humans (8–10). However, due to the high incidence of adverse reactions in vaccine recipients those with pre-existing immunity to *C. burnetii*, this vaccine requires pre-vaccination skin testing and serologic screening for existing immunity to *C. burnetii* to avoid vaccination induced adverse reactions (9). Major reason for the side effect is the pre-exposure of subjects to *C. burnetii* infection, which results in strong inflammatory response at the site of vaccination. Due to the safety concerns, this vaccine is not approved by the FDA and there is currently no licensed vaccine available for the prevention of human Q fever in the US. Therefore, there is an urgent need to develop a safe and effective vaccine for the prevention of human Q fever. However, the mechanism of protective immunity to *C. burnetii* infection is not well studied. Understanding the mechanism of host immune responses to *C. burnetii* infection will provide novel information for developing a safe and effective vaccine against human Q fever.

Previous studies have demonstrated that both humoral and cellular immune responses are required for host defense against primary *C. burnetii* infection, while T cells are considered to play critical roles in controlling intracellular bacterial replication and elimination of the organisms in mice (11–13). The role of humoral immunity in the protection of *C. burnetii* infection was first confirmed by the observation that mixture of virulent *C. burnetii* with specific antibody (Ab) was unable to cause infection in animal models (14). *In vitro* studies also indicated that incubation of virulent *C. burnetii* bacteria with immune sera made the organisms are more vulnerable to normal polymorphonuclear leukocyte- and macrophage-mediated phagocytosis and killing (15–17). In addition, the observation that B cell deficiency in mice increased the severity of histopathological changes during *C. burnetii* infection (12) suggests that B cells may also play an important role in regulating inflammatory responses to *C. burnetii* infection in mice. However, one early observation that treatment of athymic mice with immune sera 24 h before challenge with virulent *C. burnetii* has no effect on bacterial multiplication within the spleens of the T-cell-deficient mice (18) suggests that T-cell-mediated immunity plays an essential role for the control of *C. burnetii* infection. This hypothesis was supported by a previous study (12) that demonstrated T cells and IFN-γ are essential for clearance of a primary *C. burnetii* infection in mice. Additionally, it has been reported that either CD4^+^ or CD8^+^ T cells are able to control a primary pulmonary *C. burnetii* infection, suggesting that either CD4^+^ or CD8^+^ T cells are required for host defense against a primary *C. burnetii* infection in mice (11). Collectively, these findings indicate that T-cell mediated immunity may be the essential mechanism for host defense against primary *C. burnetii* infection in mice. However, it has not been investigated whether primary *C. burnetii* infection induces protection against *C. burnetii* reinfection in mice. Although a previous study (19) suggested that individuals who recovered from *C. burnetii* natural infection may acquire lifelong protective immunity against *C. burnetii* reinfection, it remains unclear which immune components are responsible for primary *C. burnetii* infection induced long-term protective immunity against *C. burnetii* reinfection. Since *C. burnetii* natural infection has the potential to elicit optimal protections against *C. burnetii* re-exposure, understanding the mechanism of *C. burnetii* infection derived protection may provide valuable insights for designing an improved new generation vaccine that can mimic *C. burnetii* natural infection.

In this study, we examined whether virulent NMI infection induces protection against *C. burnetii* reinfection in mice. Subsequently, we investigated the mechanisms of primary NMI-infection induced protective immunity against NMI reinfection in mice. This study provided the first evidence to demonstrate that primary *C. burnetii* infection provides significant protection against virulent *C. burnetii* challenge and that primary *C. burnetii* infection derived protection depends on both B and T cells, but T cells play a critical role in mice.

## Materials and methods

### Animals

All mice used in this study were female at eight to ten weeks old. BALB/c (Strain # 000651), C57BL/6 (Strain # 000664), B-cell KO (*Ighm^tm1Cgn/J^*, strain # 002288) mice, T-cell KO (*Foxn1^nu^*, strain # 000819) mice, CD4^+^ T-cell KO (*Cd4^tm1Mak^*, strain # 002663) and CD8^+^ T-cell KO (*Cd8a^tm1Mak^*, strain # 002665) mice were purchased from the Jackson Laboratory (Bar Harbor, ME). All mice were maintained in autoclaved GR900 cages (TECNIPLAST S.p.A., PA) containing four to five mice per cage under specific pathogen-free conditions at the UTSA animal biosafety level-3 laboratory facility (BSL3). Animals were provided with autoclaved RO water and 5V5R (Lab Diet, Fort Worth, TX) feed ad libitum. All research involving using animals was conducted following animal care and use guidelines. All protocols that were used in this study were approved by the Animal Care and Use Committee and Institutional Biosafety Committee of the UTSA (MU-CP001). All *C. burnetii* infection experiments were conducted in a BSL3 laboratory at the UTSA.

### Bacteria

*C. burnetii* Nine Mile Phase I (NMI) clone 7 (RSA 493) strain was cultured in acidified citrate cysteine medium-D (ACCM-D) as described previously (20). Bacteria were purified by centrifuging at 15,000 × *g* for 30 min, washed once with sterile phosphate buffer saline (PBS) and then resuspended in PBS, and stored at -80°C. All virulent NMI strain used in this study undergone four passages and all experiments involved in using virulent NMI strain were performed in a BSL3 laboratory at the UTSA.

### Primary *C. burnetii* NMI infection

For the NMI primary infection experiments, mice were infected with 1 × 10^4^ Genomic equivalents (GE) of NMI bacteria (If not specifically mentioned) and were used as NMI primary infection to investigate the mechanisms of NMI infection induced protective immunity. Mice mock infected with PBS were used as negative controls. Mice were also infected with 1 × 10^4^ GE of NMI bacteria via intraperitoneal (IP), intranasal (IN), intramuscular (IM), or subcutaneous (SC) routes as described previously (21), and were used to determine if the infection routes affect NMI primary infection induced protective immunity against *C. burnetii* challenge.

### *C. burnetii* challenge and necropsy

For the *C. burnetii* challenge, following NMI primary infection for 35 days, mice were IP injected with 1 × 10^7^ GE of NMI bacteria in 400μL of PBS for evaluating NMI primary infection induced protective efficacy or antibody responses against high dose of NMI challenge. Following high dose of NMI challenge, mouse body weight was measured at 0, 3, 7, 10 and 14 days post-challenge. Mice were sacrificed at 14 days post-challenge and spleens were collected and used for measuring bacterial burden in the spleen by a real-time quantitative PCR (qPCR). Splenomegaly was calculated by following formula (spleen weight/body weight) x 100. Mouse blood was collected by cardiac puncture technique, serum was separated by spinning blood at 1500 × *g* for 10 minutes at room temperature and used to measure antibody titer by ELISA. If not processed immediately, spleen and serum were stored at -20°C for further use.

### qPCR

Forty mg of spleen was homogenized in 200 μL of lysis buffer (0.5 M EDTA, 1 M Tris, 7 mg/mL glucose, 28 mg/mL lysozyme) and filtered through a 100-μm-pore-size nylon mesh to remove any connective tissue. Ten microliters of proteinase K (20 mg/ml) were added to each sample prior to incubation at 60°C for 18 h. Next, 21 μl of 10% SDS was added to samples and incubated at room temperature for 1 h. Finally, DNA was extracted using a High Pure PCR Template Preparation kit (Roche, Indianapolis, IN) as directed by the manufacturer. Bacterial burden was determined using Taqman assay by quantifying *C. burnetii com1* gene and normalized using mouse *tfrc* gene. Custom Plus TaqMan™ RNA Assay, FAM for *com1* (COM1 TaqMan fwd: 5′-AATAAAAACCTCCGCGTTGTCTT-3′) was designed and procured from Invitrogen. TaqMan™ Copy Number Reference Assay, mouse, *tfrc* (VIC) was used to quantify the *tfrc* genes in mouse genome. The experiment was conducted using an Applied Biosystems QuantStudio3 real-time PCR system. The standard curve was generated using recombinant plasmid DNA (*com1* gene ligated into pBluescript vector) and the results are represented as log_10_ *com1* gene copy number. All the experiments are performed at least in triplicates including both technical and biological replicates.

### *C. burnetii* NMI-specific ELISA

Sera from *C. burnetii* infected and uninfected control mice were used for quantification of total IgM, IgG, IgG1, IgG2a and IgG3 subclass antibodies. Briefly, microtiter plates (96-well) were coated with 100 μl of inactivated NMI antigen (0.5 μg/ml) or unlabeled anti-IgM, -IgG, -IgG1, -IgG2a, -IgG3 antibody (0.5 μg/ml, for the standard curve) (Southern Biotech, Birmingham, AL) in 0.05 M carbonate/bicarbonate coating buffer (pH 9.6) for 24 h at 4°C. Plates were blocked with 1% BSA in PBS-T buffer (0.05% Tween 20 in 1× PBS) and then incubated for 2 h with 100 μl of diluted sample serum (1:300) or serially diluted pure IgM, IgG, IgG1, IgG2a or IgG3 (Southern Biotech) at room temperature. Plates were washed four times with PBS-T buffer and then incubated with 100 μl of diluted horseradish peroxidase (HRP)-conjugated goat anti-mouse IgM, IgG, IgG1, IgG2a or IgG3 (1:4,000 to 1:5,000) (Southern Biotech) at room temperature for 1 h. Plates were washed again four times with PBS-T, followed by the addition of 200 μl of 3,3’,5,5’-tetramethylbenzidine (TMB) substrate (ThermoFisher Scientific, Cat# P9187). Reactions were stopped using 1 M H_3_PO_4_, and absorbance was measured at 450 nm using an Infinite F50 (Tecan, Switzerland) microplate reader.

### Flow cytometry

Mice spleens were collected after necropsy, homogenized, filtered with a 100 μm-pore-size nylon mesh to prepare a single-cell suspension, and then the centrifuged at 500 × *g* for 5 mins. Lysis of Red Blood Cells was performed by incubation of cell pellets in 5 ml of ammonium chloride-potassium (ACK) lysis buffer for 5 min at RT. After centrifugation, cell pellets were resuspended in fluorescence-activated cell sorting (FACS) buffer [PBS supplemented with 0.5% bovine serum albumin (BSA), 2mM EDTA, and 0.1% sodium azide]. Cells were incubated with Fc Block antibody (Biolegend, San Diego, CA) at 4°C for 15 min and then incubated with antibody cocktail (Biolegend) [Panel-1 (CD11c-PE, CD11b-FITC, LY6G-APC-Cy7) and Panel-2 (CD8-FITC, CD19-PE, CD3-PE-Cy7, CD4-APC-Cy7)] in FACS buffer at 4°C for 30 mins. After incubation, cells were centrifuged at 500 × *g* for 5 mins, washed twice with cold FACS buffer, and then fixed by incubating with a freshly prepared 2% paraformaldehyde solution at 4°C for 15 min. Following fixation, cells were centrifuged at 500 × *g* for 5 mins and then resuspended in FACS buffer for flow cytometry analysis. Fluorescence minus one (FMO) was used as control to determine the gates for respective cell populations in flow cytometry. Single staining of each fluorochrome with splenocytes was also used in compensation and the compensation was determined by using FlowJo software to ensure accurate separation of fluorescence signals. MoFlo XDP was used to collect cellular events. FlowJo (version 10.4.2) was used for data analysis. Absolute cell numbers were determined based on live-cell frequency and the total number of splenocytes recovered from each mouse.

### Passive transfer of immune sera

Serum samples were collected from 1 × 10^4^ GE of NMI bacteria infected or PBS control mice at 35 days post-infection and transferred to naive mice, respectively, as described previously (21). Following serum transfer for 24 hrs, mice were challenged with 1 × 10^7^ GE of virulent NMI bacteria by IP injection. Mice were sacrificed at 14 days post challenge. The protective efficacy of immune serum against NMI challenge was evaluated by comparing body weight lost, splenomegaly and bacterial burden in the spleen with controls.

### Statistical analysis

Statistical analysis was performed using Prism 9.00 (GraphPad Software Inc., San Diego, CA). One-way ANOVA with Tukey’s post-test was used to analyze the significance in the experiments involving more than two experimental groups. A two-tailed Student *t-*test was used to compare the significance between different immunization groups. For all analyses, *P* value <0.05 was considered significant.

## Results

### NMI primary infection elicited a dose independent protection against NMI reinfection

**Figure 1:**
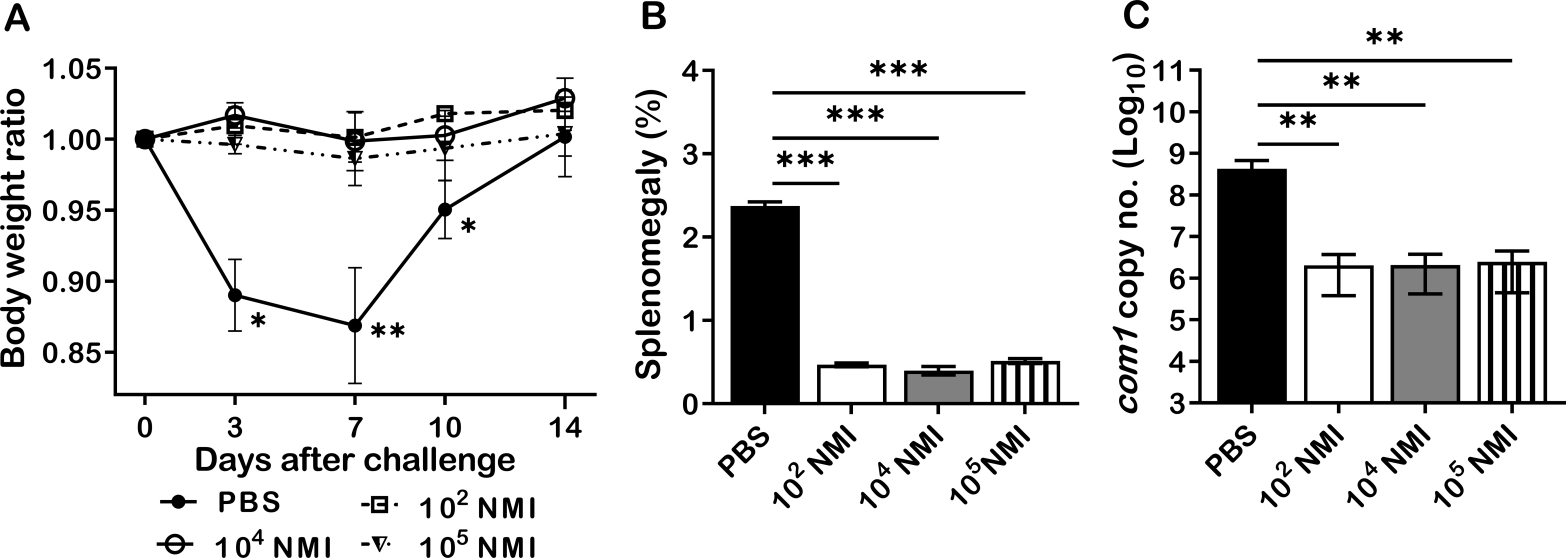
NMI primary infection elicited a dose independent protection against NMI reinfection. BALB/c mice were IP infected with PBS, 1 × 10^2^, 1 × 10^4^, or 1 × 10^5^ GE of NMI bacteria. All dose of NMI-infected and PBS control mice were IP challenged with 1 × 10^7^ GE of NMI bacteria at 35 days post primary NMI infection. Splenomegaly and bacterial burden in the spleen were measured at 14 days post-challenge. **(A)**, Relative body weights (current body weight/day 0 body weight) were measured throughout the challenge period. **(B)**, Splenomegaly (% of spleen weight/body weight). **(C)**, bacterial burden in the spleen was determined by real-time qPCR and is expressed as log10 *C. burnetii com1* gene copy numbers. Data presented in each group are the averages with standard deviations (SD) for four mice, with error bars representing the SD from the means. *P < 0.05; **P < 0.01; and ***P < 0.001 were determined by one-way ANOVA with Tukey’s post-test.

It has been documented that individuals who recovered from *C. burnetii* natural infection may acquire lifelong protective immunity against *C. burnetii* reinfection (19). However, the mechanism of *C. burnetii* natural infection derived protection against *C. burnetii* reinfection remains unexplored (22, 23). To investigate whether primary *C. burnetii* infection confers protection against secondary *C. burnetii* infection in mice, 4 groups of BALB/c mice were IP infected with PBS (mock infection control), 1 × 10^2^, 1 × 10^4^, or 1 × 10^5^ GE of virulent *C. burnetii* NMI and then all mice were challenged with a high dose, 1 × 10^7^ GE of NMI bacteria at 35 days post-primary NMI infection. Splenomegaly and bacterial burden in the spleen were measured at 14 days post- challenge. As shown in **Figure 1A**, compared to PBS control mice, all doses of NMI-infected mice protected the high dose of NMI-challenge induced transitional body weight loss at 3, 7, and 10 days post NMI-challenge. In addition, splenomegaly (**Figure 1B**) and bacterial burden (**Figure 1C**, the *com1* gene copy number) in the spleens were significantly reduced in all doses of NMI-infected mice regardless of the dose of primary infection. Collectively, these results demonstrate that primary NMI infection was able to elicit a dose independent significant protection against NMI reinfection in mice.

### NMI primary infection prevents *C. burnetii* reinfection-induced innate and adaptive cellular responses in mice

**Figure 2:**
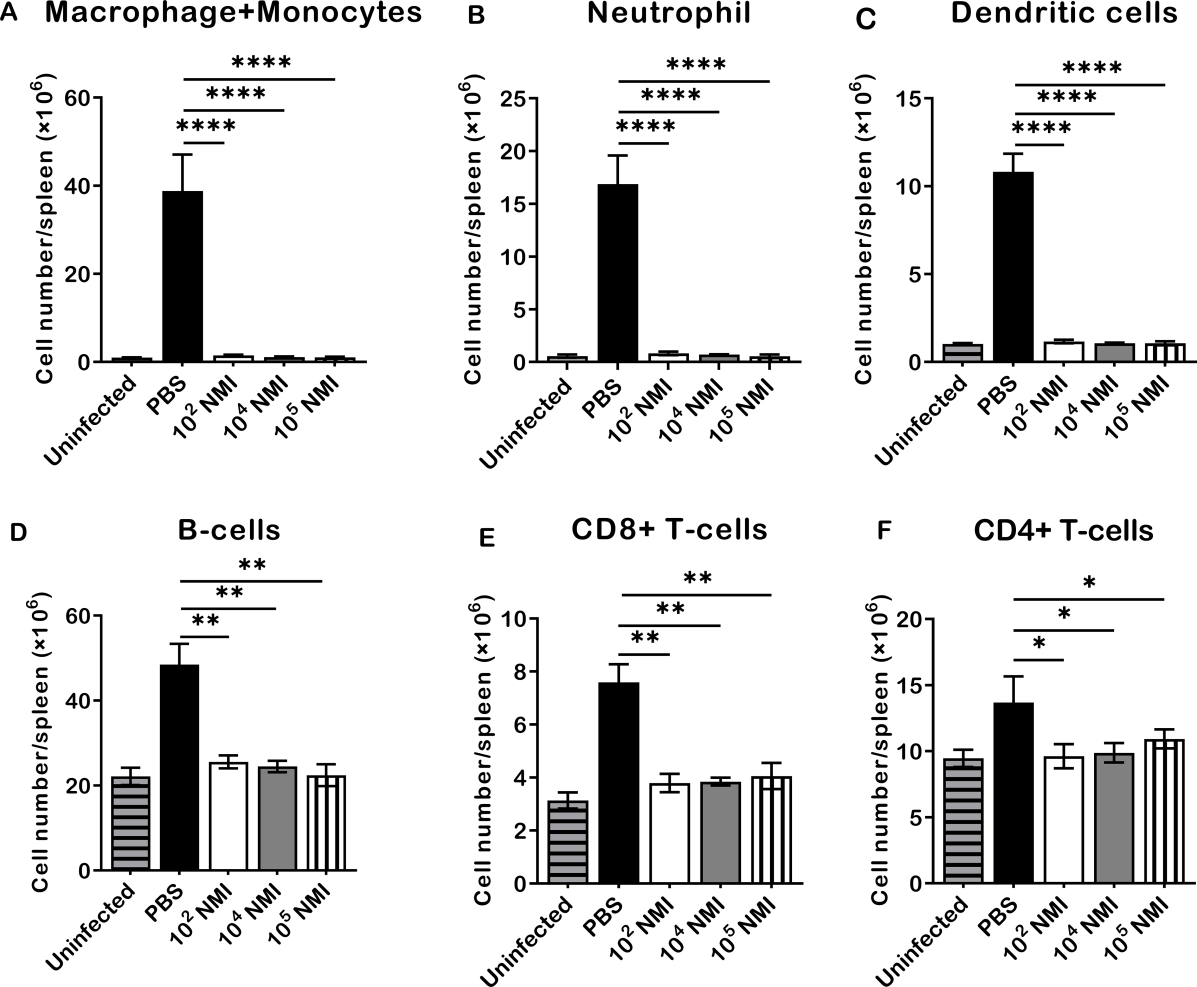
Compare NMI reinfection induced innate and adaptive cellular responses between PBS and different doses of primary NMI-infected mice. BALB/c mice were IP infected with PBS, 1 × 10^2^, 1 × 10^4^, or 1 × 10^5^ GE of NMI bacteria. All dose of NMI-infected and PBS control mice were IP challenged with 1 × 10^7^ GE of NMI bacteria at 35 days post primary NMI infection. In addition, naive BALB/c mice were used as uninfected normal mouse controls. The absolute cell numbers of macrophages plus monocytes, neutrophils, dendritic cells, B cells, CD8^+^ T cells, and CD4^+^ T cells in the spleen were determined by flow cytometry and compared between PBS control and primary NMI-infected mice at 14 days after NMI reinfection. **(A)**, macrophages plus monocytes (CD11b^+^CD11c^−^Ly6G^−^). **(B)**, neutrophils (CD11b^+^Ly6G^+^). **(C)**, Dendritic cells (CD11c^+^). **(D)**, B cells (CD19^+^). **(E)**, CD8^+^ T cells (CD3^+^CD8^+^). **(F)**, CD4^+^ T cells (CD3^+^CD4^+^). Data presented in each group are the averages with SD for four mice, with error bars representing the SD from the means. *P < 0.05; **P < 0.01 and ****P < 0.0001 were determined by one-way ANOVA with Tukey’s post-test.

To evaluate *C. burnetii* reinfection induced innate and adaptive cellular responses in mice, the total numbers of macrophages plus monocytes (CD11b^+^CD11c^−^Ly6G^−^), neutrophils (CD11b^+^Ly6G^+^), dendritic cells (CD11c^+^), B cells (CD19^+^), CD8^+^ T cells (CD3^+^CD8^+^), and CD4^+^ T cells (CD3^+^CD4^+^) in the spleens were determined by flow cytometry analysis as described in **Supplementary Figure S1**, and compared between PBS control and different doses of primary NMI-infected mice at 14 days after the high dose of NMI reinfection. As shown in **Figure 2**, compared to PBS control mice, significant lower numbers of macrophages and monocytes (**Figure 2A**, P<0.0001), neutrophils (**Figure 2B**, P<0.0001), dendritic cells (**Figure 2C**, P<0.0001), B cells (**Figure 2D**, P<0.01), CD8^+^ T cells (**Figure 2E**, P<0.01), and CD4^+^ T cells (**Figure 2F**, P<0.05) were detected in the spleens from primary NMI-infected mice in a dose independent manner. These results demonstrate that primary NMI-infected mice were able to control *C. burnetii* reinfection-induced innate and adaptive cellular responses, suggesting that primary NMI infection can elicit protective immunity in mice against *C. burnetii* reinfection, which enable primary NMI-infected mice to control bacterial replication and *C. burnetii* reinfection-induced inflammatory responses. These results also provide additional evidence to explain why primary NMI-infected mice had significant lower splenomegaly and bacterial burden in the spleen at 14 days post-NMI reinfection (**Figures 1B, C**).

### Antibody responses in primary NMI-infected mice against NMI reinfection

**Figure 3:**
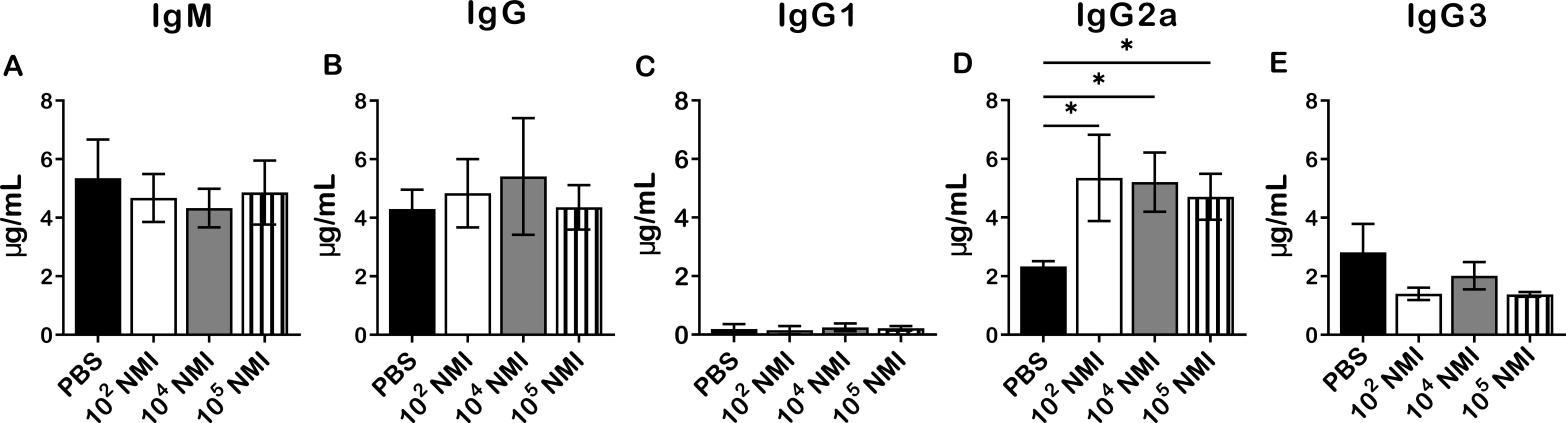
Compare NMI reinfection induced Ab responses between PBS and different doses of primary NMI-infected mice. The concentrations of anti-NMI specific IgM **(A)**, IgG **(B)**, IgG1 **(C)**, IgG2a **(D)**, and IgG3 **(E)** in immune sera from PBS or different doses of NMI primary infected mice and challenged with NMI at 14 days post-challenged were determined by ELISA. Ab concentration is expressed as µg/ml. Data presented in each group are the averages with SD for four mice, with error bars representing the SD from the means. *P < 0.05 were determined by one-way ANOVA with Tukey’s post-test.

To analyze antigen-specific antibody responses in primary NMI-infected mice against NMI reinfection, immune sera were collected from mice IP infected with PBS, 1 × 10^2^, 1 × 10^4^, or 1 × 10^5^ GE of NMI at 14 days post-challenged with 1 × 10^7^ GE of NMI bacteria in the above experiment for measuring the concentrations of anti-NMI specific IgM and IgG by ELISA. As shown in **Figure 3**, there was no significant difference in the concentrations of NMI-specific IgM (**Figure 3A**), IgG (**Figure 3B**), IgG1 (**Figure 3C**) and IgG3 (**Figure 3E**) between PBS and NMI infected mice at 14 days post-reinfection with NMI. However, the concentrations of NMI-specific IgG2a in all doses of primary NMI-infected mice were significantly higher than the IgG2a concentrations in PBS control mice at 14 days post-reinfection with NMI (**Figure 3E**). These results indicate that primary NMI infection induces higher numbers of NMI-specific IgG2a producing memory B cells, which rapidly response to NMI reinfection leading to production of significant higher IgG2a in NMI-infected mice than in PBS infected mice. This observation also suggests that NMI-specific IgG2a may play an important role in primary NMI infection induced protective immunity against NMI reinfection.

### Immune sera from primary NMI*-*infected mice provided significant protection against *C. burnetii* infection

**Figure 4:**
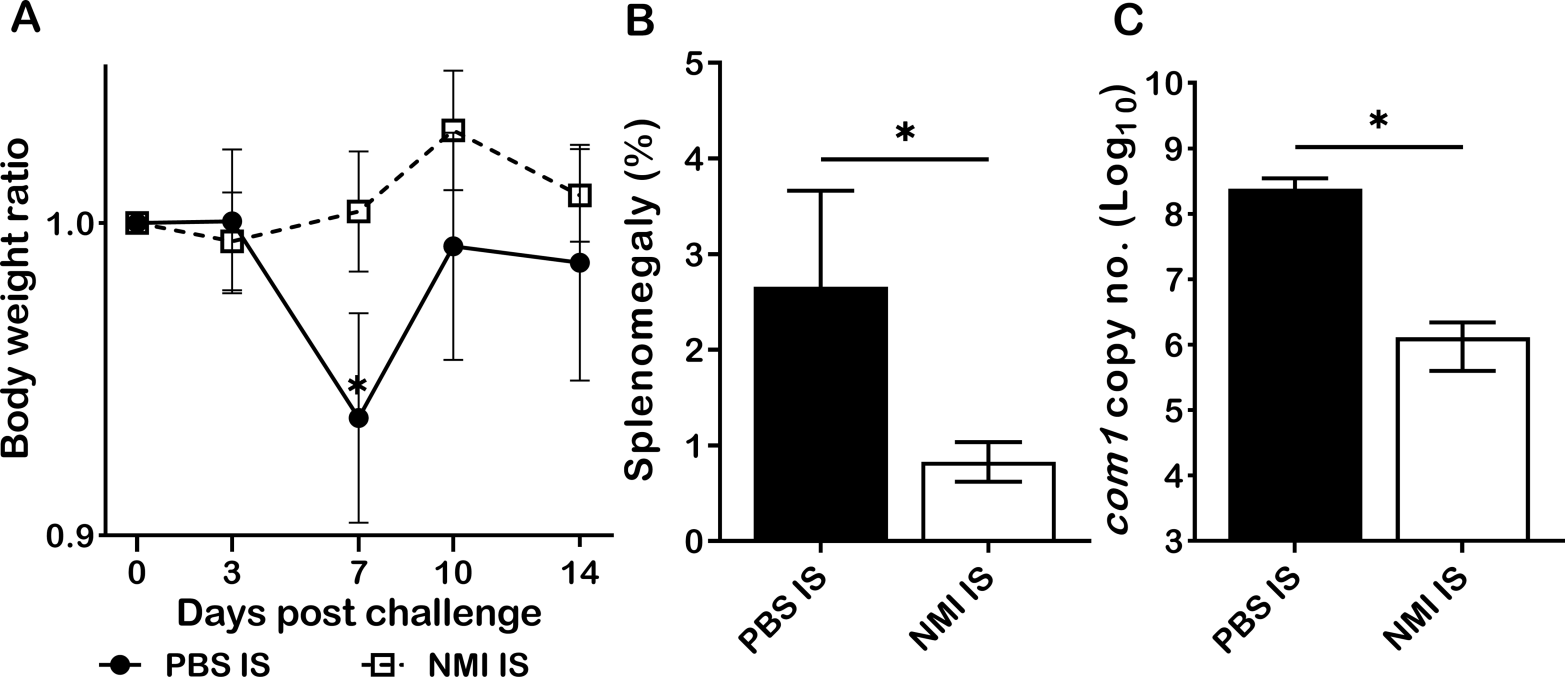
Immune sera from primary NMI*-*infected mice provided significant protection against *C. burnetii* NMI infection in naive mice. BALB/c mice receiving sera from PBS or primary NMI-infected mice were challenged with 1x10^7^ GE of NMI bacteria. Splenomegaly and bacterial burden in the spleen were measured at 14 days post-challenge. **(A)**, Relative body weights (current body weight/day 0 body weight) were measured throughout the challenge period. **(B)**, Splenomegaly (% of spleen weight/body weight). **(C)**, bacterial burden in the spleen was determined by real-time qPCR and is expressed as log10 *C. burnetii com1* gene copy numbers. Data presented in each group are the averages with SD for four mice, with error bars representing the SD from the means. *P < 0.05 were determined by one-way ANOVA with Tukey’s post-test **(A)**, unpaired Student’s *t*-test **(B, C)**.

The observation that either CD4^+^ or CD8^+^ T cells from primary *C. burnetii* infected mice are able to provide protection against *C. burnetii* pulmonary infection suggests that either CD4^+^ or CD8^+^ T cells are required for primary *C. burnetii* infection induced protective immunity (11). However, it remains uninvestigated if humoral immunity contributed to primary *C. burnetii* infection-induced protection against *C. burnetii* reinfection. To determine if passive transfer of immune sera from primary NMI-infected mice will provide significant protection to naive recipient mice against *C. burnetii* infection, immune sera were collected from mice IP infected with PBS or 1 × 10^4^ GE of NMI bacteria at 35 days post-infection and transferred to naive mice, respectively. Following transfer of immune sera for 24 hrs, immune sera recipient mice were challenged with 1 × 10^7^ GE of NMI bacteria. As shown in **Figure 4A**, compared to mice receiving sera from PBS injected control mice, mice receiving immune sera from NMI-infected mice protected the high dose of NMI-infection induced transitional body weight loss at 7 days post NMI-infection. In addition, splenomegaly (**Figure 4B**) and bacterial burden (**Figure 4C**, the *com1* gene copy number) in the spleens were significantly reduced in mice receiving immune sera from NMI-infected mice. These results indicate that immune sera from NMI-infected mice provided significant protection against high dose NMI infection, suggesting that *C. burnetii*-specific antibodies may play important roles in primary NMI infection-induced protective immunity.

### Both B cells and T cells are required for primary NMI-infection induced protective immunity, but T cells may play a critical role in controlling bacterial replication

**Figure 5:**
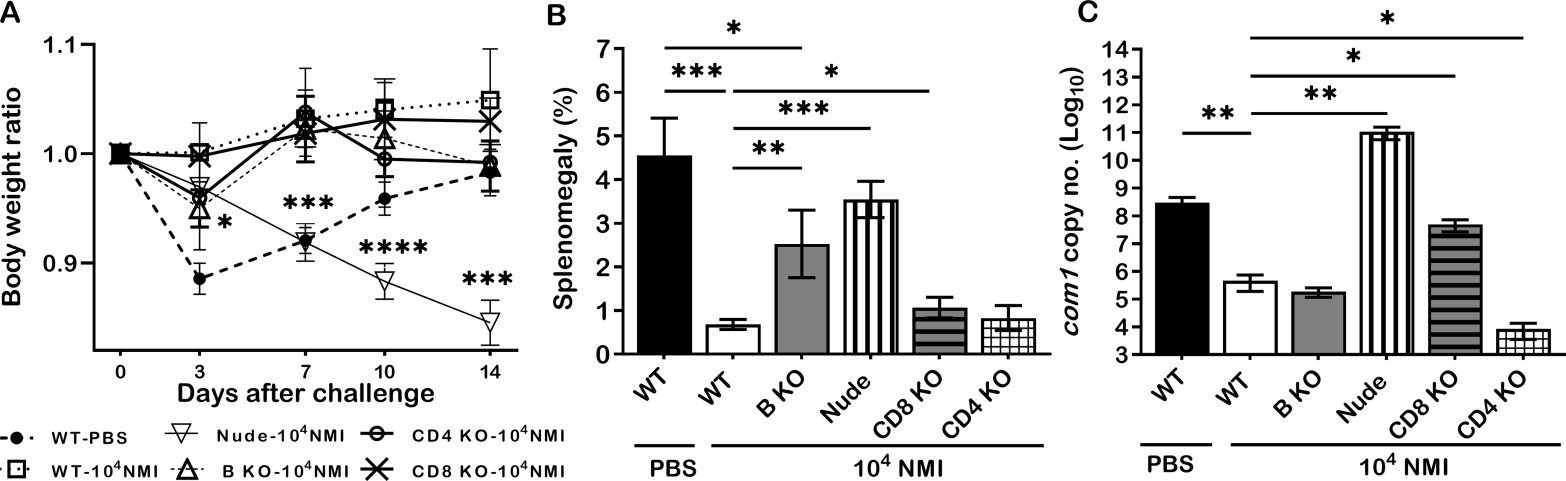
The protective efficacy of primary NMI-infection induced protection against NMI reinfection in B cell, T cell, CD4^+^ T cell or CD8^+^ T cell deficient mice. C57BL/6J WT and B cell, T cell, CD4^+^ T cell or CD8^+^ T cell deficient mice were IP infected with 1 × 10^4^ GE of NMI bacteria and challenged with 1 × 10^7^ GE of NMI bacteria at 35 days post-primary NMI-infection. Additionally, naive WT mice were mock infected with PBS and served as controls. Splenomegaly and bacterial burden in the spleen were measured at 14 days post-challenge. **(A)**, Relative body weights (current body weight/day 0 body weight) were measured throughout the challenge period. **(B)**, Splenomegaly (% of spleen weight/body weight). **(C)**, bacterial burden in the spleen was determined by real-time qPCR and is expressed as log10 *C*. *burnetii com1* gene copy numbers. Data presented in each group are the averages with SD for five mice, with error bars representing the SD from the means. *P < 0.05; **P < 0.01; ***P < 0.001; and ****P < 0.0001 were determined by one-way ANOVA with Tukey’s post-test.

To further understand the mechanisms of primary NMI-infection induced protective immunity, we examined if B cell, T cell, CD4^+^ T cell or CD8^+^ T cell deficiency in mice will significantly affect primary NMI-infection induced protective immunity against NMI reinfection. C57BL/6J WT and B cell, T cell, CD4^+^ T cell or CD8^+^ T cell deficient mice were IP infected with 1 × 10^4^ GE of NMI bacteria and challenged with 1 × 10^7^ GE of NMI bacteria at 35 days post-primary NMI-infection. Additionally, naive WT mice were mock infected with PBS and served as controls. Splenomegaly and bacterial burden in the spleen were examined at 14 days post-challenge. As shown in **Figure 5A**, NMI-infection induced a significant bodyweight loss in NMI-infected B cell deficient and T cell deficient mice at different times post-challenge but there was no significant bodyweight loss in PBS-infected WT, and NMI-infected WT, CD4^+^ T cell deficient and CD8^+^ T cell deficient mice. In addition, compared to PBS control WT mice, splenomegaly was significantly reduced in all primary NMI-infected mice (**Figure 5B**). However, although splenomegaly was similar between primary NMI-infected B cell deficient and T cell deficient mice, it was significantly higher than NMI-infected WT, CD4^+^ T cell deficient and CD8^+^ T cell deficient mice. As shown in **Figure 5C**, the bacterial burden was comparable in the spleens from primary NMI-infected WT, B cell deficient and CD4^+^ T cell deficient mice, but it was significantly higher in the spleen from primary NMI-infected CD8^+^ T cell deficient and T cell deficient mice. Notably, the bacterial burden in the spleen from primary NMI-infected T cell deficient mice even significantly higher than PBS-infected WT mice. Collectively, these results demonstrated that primary NMI infection induced a comparable level of protection against the high dose NMI reinfection in WT and CD4^+^ T cell deficient mice, and a partial protection in B cell deficient, CD8^+^ T cell deficient and T cell deficient mice. The observation that the bacterial burden in the spleens from primary NMI-infected T cell deficient mice was significantly higher than PBS-infected WT control mice suggests that T cells, especially, CD8^+^ T cells may play a critical role in controlling virulent *C. burnetii* replication in mice. Thus, primary NMI infection induced protective immunity depends on both B cells and T cells but T cells may play a critical role in controlling bacterial replication.

### *C. burnetii* NMI infection derived protection did not depend on the routes of infection

**Figure 6:**
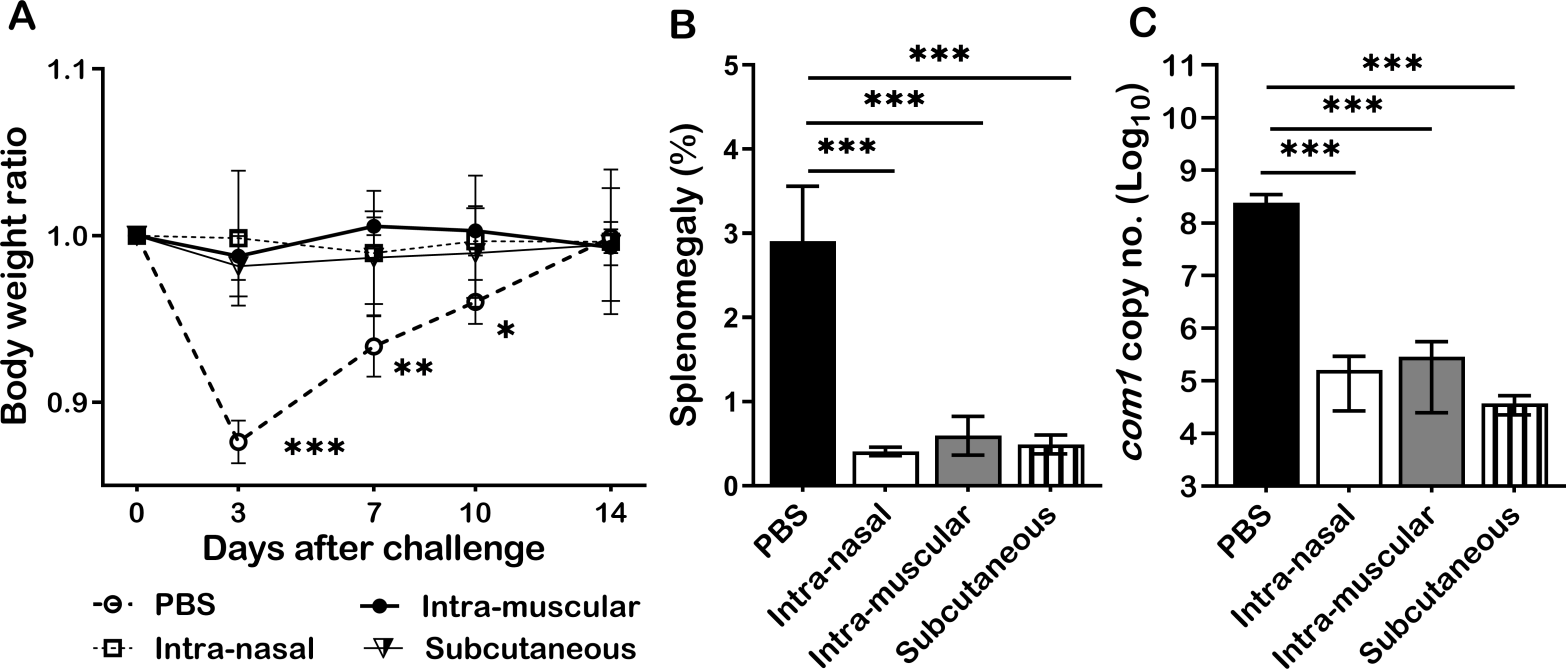
Primary NMI infection derived protection did not depend on the routes of infection. BALB/c mice were IN, IM, or SC infected with 1 × 10^4^ GE of NMI bacteria and IP challenged with 1 × 10^7^ GE of NMI bacteria at 35 days post-primary NMI-infection via different routes. In addition, PBS receiving mice were IP challenged with 1x10^7^ of NMI bacteria and used as unimmunized controls. Splenomegaly and bacterial burden in the spleen were measured at 14 days post NMI challenge. **(A)**, Relative body weights (current body weight/day 0 body weight) were measured throughout the challenge period. **(B)**, Splenomegaly (% of spleen weight/body weight). **(C)**, bacterial burden in the spleen was determined by real-time qPCR and is expressed as log10 *C. burnetii com1* gene copy numbers. Data presented in each group are the averages with SD for five mice, with error bars representing the SD from the means. *P < 0.05; **P < 0.01; and ***P < 0.001 were determined by one-way ANOVA with Tukey’s post-test.

To examine whether infection route would affect primary NMI infection derived protection against NMI reinfection, mice were IN, IM, or SC infected with 1 × 10^4^ GE of NMI bacteria and IP challenged with 1 × 10^7^ GE of NMI bacteria at 35 days post-primary NMI-infection via different routes. In addition, PBS receiving mice were IP challenged with 1x10^7^ of NMI bacteria and used as unimmunized controls. Splenomegaly and bacterial burden in the spleen were measured at 14 days post NMI challenge. As shown in **Figure 6A**, compared to PBS controls, all different routes of primary NMI-infected mice protected the high dose NMI-challenge induced transitional body weight loss. Splenomegaly (**Figure 6B**) and bacterial burden in the spleens (**Figure 6C**) were also significantly reduced in all primary NMI-infected mice regardless the route of infection. These results demonstrate that infection route did not affect NMI infection derived protection against NMI reinfection.

## Discussion

Elucidation of nature infection induced protective immune responses has provided crucial information for vaccine development against other microbial pathogens (24–26). Thus, understanding the mechanism of *C. burnetii* infection derived protection may provide valuable insights for designing an improved new generation vaccine against human Q fever. This study aimed to investigate if virulent NMI infection induces protective immunity against *C. burnetii* reinfection in mice. We found that NMI-infected mice were able to control bacterial replication and *C. burnetii* reinfection-induced inflammatory responses, suggesting that NMI-infected mice acquired protective immunity against *C. burnetii* reinfection. In addition, this study provided first evidence to demonstrate that NMI infection derived protective immunity depends on both B cells and T cells, while T cells maybe crucial for controlling bacterial replication and eliminating the bacteria. These findings provide important implications for development of new generation vaccines against human Q fever.

To understand the innate and adaptive cellular responses in primary NMI infected mice against *C. burnetii* reinfection, the total numbers of macrophages plus monocytes, neutrophils, dendritic cells, B cells, CD8^+^ T cells, and CD4^+^ T cells in the spleens were compared between PBS control and different doses of primary NMI-infected mice at 14 days post-NMI challenge. The results indicated that significant lower numbers of macrophages and monocytes, neutrophils, dendritic cells, B cells, CD8^+^ T cells, and CD4^+^ T cells were detected in the spleens from primary NMI-infected mice than in PBS control mice, suggesting that primary NMI-infected mice protected *C. burnetii* reinfection-induced innate and adaptive cellular responses. In addition, the results that primary NMI-infected mice had significant lower splenomegaly and bacterial burden in the spleen than PBS control mice at 14 days post-NMI reinfection (**Figures 1B, C**) suggest that primary NMI infection can elicit protective immunity in mice against *C. burnetii* reinfection, which enable primary NMI-infected mice to control bacterial replication and inflammatory responses against *C. burnetii* reinfection. It has been shown that host control of virulent *C. burnetii* infections and clearance of the organisms are considered to be mediated primarily by activated monocytes and macrophages (27). Our previous study also demonstrated that neutrophils play an important role in host defense against virulent *C. burnetii* infection (28). The result that significant higher numbers of neutrophils, monocytes and macrophages were detected in the spleens from PBC control mice at 14 days post-NMI infection provides additional evidence to support that neutrophils, monocytes and macrophages are crucial for host innate immune defense against virulent *C. burnetii* infection. Despite dendritic cells are the most potent antigen presenting cells (APCs) and have demonstrated their important roles in activating adaptive immune responses to eradicate microbial pathogens, the role of DCs in host defense against virulent *C. burnetii* infection *in vivo* has not been well studied. Since spleen is a major peripheral lymphoid organ for APCs to activate T and B cells, and both B cells and T cells are required for host immune defense against virulent *C. burnetii* infection (12, 29), the significantly higher numbers of DCs, B cells, and T cells in the spleens of PBS control mice suggest that DCs may play an important role in activation of T and B cells in the spleen in response to virulent *C. burnetii* infection in mice. Further studies focuses on determining the role of DCs in activation of T and B cells in mice would provide critical information for understand the mechanism of host immune defense against *C. burnetii* infection.

In general, it is considered that Ab-mediated immunity plays an essential role for host immune defense against extracellular bacterial pathogens, while cell-mediated immunity is crucial for control and clearance of intracellular bacterial pathogens. However, accumulating evidence demonstrates Abs play an important role for host defense as well as for vaccine-induced protective immune responses against various intracellular bacterial pathogens (30, 31). Despite the obligate intracellular lifestyle of *C. burnetii*, Abs have been demonstrated their important roles for host defense and vaccine-induced protection against *C. burnetii* infections in animals and humans (32). A previous study (33) reported that NMI-specific IgM Abs were detected from acute Q fever patients, while high levels of NMI-specific IgG, IgA Abs were appeared in patients with chronic Q fever. Our recent study also demonstrated that inactivated NMI-WCV immunized mice developed significantly high levels of IgM and IgG Abs (34). In the current study, we compared NMI-specific IgM, IgG, IgG1, IgG21 and IgG3 responses between PBS control and different doses of primary NMI-infected mice at 14 days post-NMI challenge. Interestingly, although the concentrations of NMI-specific IgM, IgG, IgG1 and IgG3 were similar between PBS and NMI infected mice, the concentrations of NMI-specific IgG2a in all doses of primary NMI-infected mice were significantly higher than the IgG2a concentrations in PBS control mice at 14 days post- NMI reinfection (**Figure 3**). These results suggest that primary NMI infection may elicit higher numbers of NMI-specific IgG2a producing memory B cells, which can rapidly response to NMI reinfection resulting the production of significant higher levels of IgG2a in primary NMI-infected mice than in PBS control mice. In addition, the observation that passive transfer of immune sera from primary NMI-infected mice provided significant protection to naive recipient mice against *C. burnetii* infection (**Figure 4**) suggests that NMI-specific Abs may play important roles in primary NMI infection-induced protective immunity. Since IgG2a is a marker that associated with a Th1 response in mice (35), our results suggest that primary NMI infection may be able to induce a Th1 immune response and that a Th1 immune response may be important for primary NMI infection-induced protective immunity. This hypothesis is supported by our previous report that significant higher levels of IgG2a than IgG1 were detected in unvaccinated, PIV or PIIV vaccinated mice at 14 days post-challenge with NMI (18). In addition, NMI-LPS recognizing IgG2a mAb 1E4 was demonstrated its ability to provide significant protection against *C. burnetii* infection in our previous study (36). Collectively, these data suggest that NMI-specific IgG2a may play a critical role in primary NMI infection-induced protective immunity against NMI reinfection. However, future study is needed to determine whether Th1 subset CD4^+^ T cell response is responsible for the primary NMI infection-induced NMI-specific IgG2a response.

To identify which components are responsible for the primary NMI-infection induced protection, we examined if B cell, T cell, CD4^+^ T cell or CD8^+^ T cell deficiency in mice will significantly affect primary NMI-infection induced protective immunity against NMI reinfection. Our results demonstrated that primary NMI infection induced a comparable level of protection against the high dose NMI reinfection in WT and CD4^+^ T cell deficient mice, and a partial protection in B cell deficient, CD8^+^ T cell deficient and T cell deficient mice. In addition, the bacterial burden in the spleens from primary NMI-infected T cell deficient mice was significantly higher than PBS-infected WT control mice. Thus, primary NMI infection induced protective immunity may depend on both B cells and T cells but T cells, especially, CD8^+^ T cells may play a critical for controlling bacterial replication as well as for clearance of bacteria. Our previous studies have also demonstrated that both B cells and T cells are required for viable NMII vaccine (21) and formalin-inactivated-NMI vaccine (29)-induced protective immunities against virulent *C. burnetii* NMI challenge but T cells are crucial for controlling bacterial replication and for clearance of bacteria. These data suggest that the mechanism of primary *C. burnetii* infection-induced protection against *C. burnetii* reinfection maybe similar to the mechanisms of viable NMII vaccine and inactivated-NMI vaccine-induced protection and highlight that T cells play a critical role for controlling bacterial replication in protective immunity against this intracellular bacterial pathogen.

It is notable that primary NMI infected B cell deficient mice were unable to prevent high dose NMI challenge induced body weight loss and splenomegaly but protected NMI challenge induced bacterial burden in the spleens. These results suggest that B cells may play a crucial role in protecting NMI challenge induced clinical diseases and inflammatory responses in primary NMI infected mice. This hypothesis is supported by a previous study (10) demonstrating that B cell deficiency in mice increases the severity of histopathological changes during virulent *C. burnetii* NMI primary infection but did not affect the bacterial burden in spleens and our previous report that showed B1a cells secreted a high level of anti-inflammatory cytokine, IL-10 in response to *C. burnetii* infection *in vitro* (37). These data suggest that B cells may play an important role in regulating host inflammatory responses against *C. burnetii* infection in mice. Additionally, Th1 immune response and IFN-γ have been demonstrated to be critical for host defense against *C. burnetii* primary infection (10) as well as for vaccine-induced protective immunity against *C. burnetii* infection (18, 38). However, it has been shown that uncontrolled activation of Th1 cells can cause host tissue damage during persistent bacterial infections (39). B cells have been demonstrated their ability to produce IL-10 in response to microbial infections (40) and IL-10 negatively regulates activated Th1 cells to secret IFN-γ, which is crucial for maintaining a Th1/Th2 balance response during microbial infections (41). In addition, our previous study also demonstrated that adoptive transfer of B cells protects B cell deficient mice from weight loss and reduces serum IFN-γ levels during NMI infection (37). Thus, it is possible that B cells may play an important role in maintaining a balanced Th1/Th2 immune response during *C. burnetii* infection. Future studies to investigate if B cells can directly influence Th1 and Th2 immune responses in both *in vitro* and *in vivo* systems would be helpful to determine whether B cells may play an important role in regulating T cell-mediated immunity during *C. burnetii* infection.

The results that primary NMI infected T cell deficient mice were unable to prevent high dose NMI challenge induced body weight loss, splenomegaly and bacterial burden in the spleens indicate T cells play a major role for primary NMI infection-induced protective immunity. A previous study has been shown that although either CD4^+^ or CD8^+^ T cells were able to effectively control virulent *C. burnetii* primary infection, CD8^+^ T cells were more critical than CD4^+^ T cells (11). Our previous study demonstrating that MHC-I deficient mice developed more severe diseases than MHC-II deficient mice, which supports the hypothesis that CD8^+^ T cells play a more critical role than CD4^+^ T cells in host defense against NMI primary infection (13). The current results that the bacterial burden was similar in the spleens from primary NMI-infected WT, B cell deficient and CD4^+^ T cell deficient mice, but it was significantly higher in the spleen from primary NMI-infected CD8^+^ T cell deficient and T cell deficient mice (**Figure 5C**), suggest that CD8^+^ T cells may play a critical for controlling bacterial replication as well as for clearance of bacteria in primary NMI-infected mice against NMI reinfection. This hypothesis is also supported by our previous study demonstrating that MHC-I restricted CD8^+^ T cells play a more critical role in controlling bacterial replication during primary *C. burnetii* infection and that perforin, but not granzyme B, is required for cytotoxic CD8^+^ T cells to control primary *C. burnetii* infection (13). However, it remains unknown if NMI primary infection would be able to elicit *C. burnetii*-specific T-cell memory responses. Future study to directly examine whether NMI primary infection can induce stronger *C. burnetii*-specific T-cell memory responses than PIV vaccination would provide crucial insights for the development of safe and effective new generation vaccines against human Q fever.

*C. burnetii* natural infections in humans commonly occur via inhalation of infectious aerosols generated from *C. burnetii* infected animal feces, urine, milk, and birth products (42). However, due to ease of handling and a lack of aerosol instrumentation, *C. burnetii* IP infection animal models have been commonly used for studying host immune responses against primary *C. burnetii* as well as for understanding the mechanisms of vaccine-induced protective immunity. Our previous studies (16, 27, 28, 33, 34) have also demonstrated that both *C. burnetii* IP and IN infections reliably induced significant splenomegaly in mice and that splenomegaly correlates to infection dose, bacterial burden and pathological changes in the spleen, suggesting that splenomegaly and bacterial burden in the spleen can be reliable and useful parameters to monitor the severity of *C. burnetii* infection in mice. In this study, the *C. burnetii* IP infection mouse model was used to understand the mechanism for primary *C. burnetii* infection-induced protection against *C. burnetii* reinfection. However, it is unclear if the routes of infection would affect primary NMI-infection induced protection against a high dose of NMI challenge. To address this question, we examined whether mice primary infection with 1 × 10^4^ GE of NMI bacteria by IN, IM, or SC route would influence their ability to confer protection against IP challenged with 1 × 10^7^ GE of NMI bacteria. The observation that all different routes of primary NMI-infected mice protected the high dose NMI-challenge induced transitional body weight loss, splenomegaly, and bacterial burden in spleens (**Figure 6**) suggests that primary NMI infection derived protection against NMI reinfection is infection route independent.

Despite the fact that Q-VAX has successfully reduced the spread of Q fever among occupational risk groups in Australia since the implementation of a national Q fever management program in 2001 (43), it has failed to gain approval by the FDA due to adverse reactions associated with pre-existing immunity. Therefore, understanding which vaccine components elicit the hypersensitivities, as well as the mechanisms of protective immunity, is necessary to rationally design a safe and effective Q fever vaccine. This study reveals *C. burnetii* primary infection-driven mechanisms of protective immunity can be integrated with our previously characterized mechanisms of viable NMII vaccine (21) and formalin-inactivated-NMI vaccine (18, 29, 38)-induced protective immunity to inform future Q fever vaccine development. Specifically, the observation that primary NMI infection induces significant higher level of IgG2a response to *C. burnetii* reinfection in this study correlates with our previous finding that IgG2a was the predominant IgG isotype in PIV-vaccinate mice against *C. burnetii* challenge (18), suggesting that *C. burnetii*-specific IgG2a may be an important protective component against *C. burnetii* infection. In addition, the role of CD8^+^ T cells for controlling bacterial replication as well as for clearance of bacteria was highlighted in the *C. burnetii* primary infection (current study), the viable NMII vaccine (21) and the formalin-inactivated-NMI vaccine (29)-induced protective immunity against *C. burnetii* infection. Thus, antigens that activate B cells to produce *C. burnetii*-specific IgG2a as well as peptide antigens that activate CD8^+^ T cells are expected to be promising candidates for a defined vaccine against *C. burnetii* infection. However, the finding that primary NMI infection induced a comparable level of protection against the high dose NMI reinfection in WT and CD4^+^ T cell deficient mice was supported by our previous studies by demonstrating that the viable NMII vaccine (21) and the formalin-inactivated-NMI vaccine (29) also provided a similar level of protection in WT and CD4^+^ T cell deficient mice. This data suggests that *C. burnetii* primary infection and vaccine-induced protective immunity maybe independent on CD4^+^ T cells. On the other hand, a recent study (44) demonstrates that the formalin-inactivated *C. burnetii* phase I whole cell vaccine induces a Th1 delayed-type hypersensitivity (DTH) response in a sensitized mouse model, which mediated by IFNg+ and IL17a+ CD4^+^ T cells. DTH reactions occur when CD4^+^ T cells recognize foreign antigens via MHC-II-restricted antigen presentation and consequently stimulate cytokine-mediated inflammation (45). Thus, CD4^+^ T cells may be responsible for the Q-VAX-induced adverse reactions, future Q fever vaccines should modulate the CD4^+^ T cell response to reduce inflammation without sacrificing protection.

## Data Availability

The raw data supporting the conclusions of this article will be made available by the authors, without undue reservation.
