# Supplementary material for: Coxiella burnetii Nine Mile phase I primary infection derived protective immunity against C. burnetii reinfection in mice depends on both B and T cells, but T cells play a critical role
Source: Front Immunol. 2024 Oct 14;15:1427822. doi: 10.3389/fimmu.2024.1427822 (PMC11513344; doi:10.3389/fimmu.2024.1427822)
Supplement: Supplementary file 1 [file DataSheet1.pdf]

Flow cytometry analysis gating strategy for figure 2

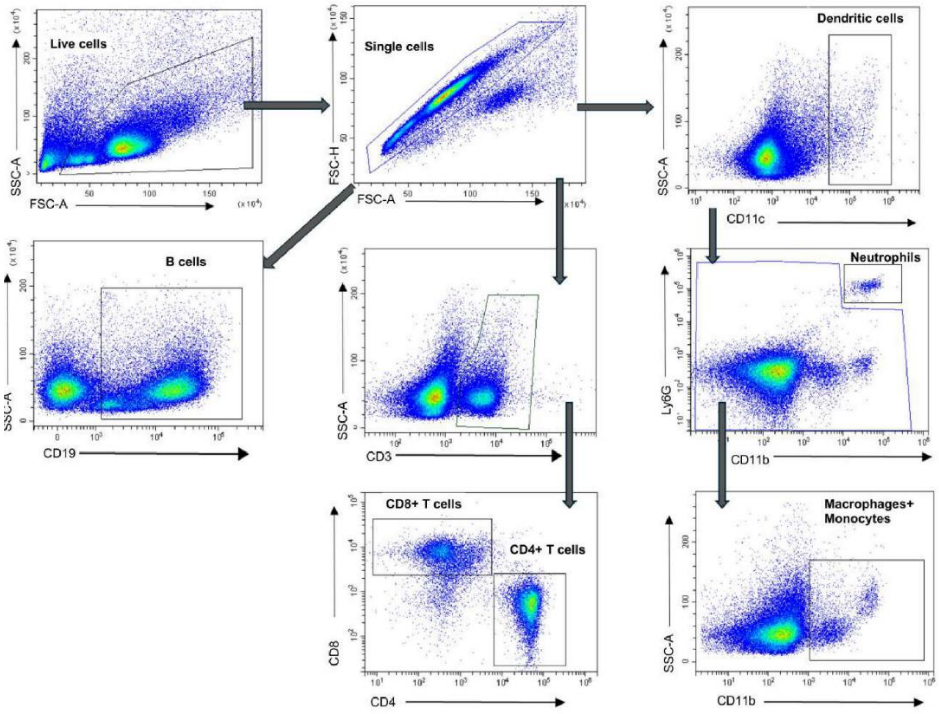

Figure S1: Representative flow cytometry gating strategy for figure 2. Live cells were identified based on forward scatter (FSC) and side scatter (SSC) properties. Single cells were then gated using FSC-Height (FSC-H) vs FSC-Area (FSC-A) to exclude doublets. From the single cell population, various immune cell subsets were identified sequentially: dendritic cells ($CD11c^+$), B cells ($CD19^+$), and T cells ($CD3^+$), further subdivided into $CD8^+$ and $CD4^+$ T cells. From the non-dendritic cell population, neutrophils were identified ($CD11b^+$ $Ly6G^+$). The remaining non-neutrophil cells were then gated for $CD11b^+$ cells to identify macrophages and monocytes. Plots show representative data with sequential gating hierarchy indicated by arrows. FSC: Forward Scatter; SSC: Side Scatter; A: Area; H: Height.
